# Tumor redox heterogeneity-responsive nanoparticles for enhanced antitumor efficacy through combining chemo/chemodynamic therapy

**DOI:** 10.1016/j.ijpx.2025.100455

**Published:** 2025-11-24

**Authors:** Su Cui, Li Yu, Hao Liu, Wenhan Liu, Daiwang Shi

**Affiliations:** a Department of Thoracic Surgery, Shengjing Hospital of China Medical University, Shenyang 110004, China; b Department of Thoracic Surgery, The First Hospital of China Medical University, Shenyang 110002, China; c Department of Oncology, Shengjing Hospital of China Medical University, Shenyang 110004, China; d Department of Obstetrics and Gynecology, Shengjing Hospital of China Medical University, Shenyang 110004, China

**Keywords:** Cancer, Nanoparticle, Redox Heterogeneity-Responsive, Chemo/Chemodynamic Therapy

## Abstract

Cancer represents a significant global health threat, and traditional chemotherapy (CT) often encounters limitations in efficacy due to systemic toxic side effects and tumor heterogeneity. The combination of chemodynamic Therapy (CDT) and CT offers a potential solution to overcome the constraints of single-agent therapies. However, many CT/CDT collaborative systems have critical shortcomings, including insufficient active targeting capabilities, depletion of H_2_O_2_ substrates leading to a reduction in CDT effectiveness, and the heterogeneity of redox within tumor cells, which can result ultimately limit overall efficacy. This study developed a redox heterogeneity-responsive CT/CDT nanoparticle, named HFMD, with the goal of overcoming the limitations associated with traditional CT/CDT nanoparticles. *In vitro* experiments demonstrated that HFMD exhibits redox-sensitive drug release characteristics and the capacity to generate hydroxyl free radicals. Additionally, HFMD enhances H_2_O_2_ supply, improves CDT efficiency, and shows significant inhibitory effects on multiple cancer cell lines. *In vivo* experiments further validated that HFMD possesses excellent tumor-targeting enrichment capabilities and remarkable anti-cancer efficacy, achieving a tumor inhibition rate of approximately 80.1 %. The biological safety assessment indicated that HFMD demonstrates good biocompatibility and successfully mitigates the dose-limiting toxicity associated with free doxorubicin. Overall, this study presents a promising strategy for enhancing anti-cancer efficacy.

## Highlights

• The nanoparticle designed to harness the CT/CDT synergy promotes ferroptosis and apoptosis for cancer therapy.
• Combines redox heterogeneity responsiveness with ROS bursts within a single nanoparticle.
• The excellent therapeutic effect and biosafety of HFMD have potential clinical transformation potential.

## Graphical abstract

**Figure:**
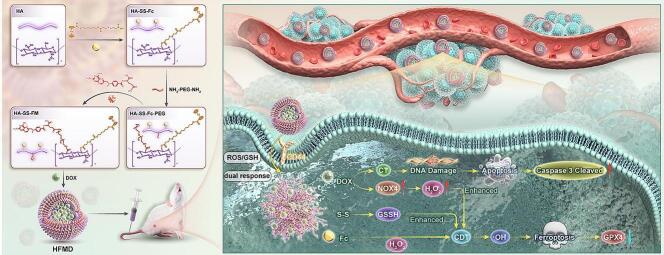

## 1 Introduction

As a major disease threatening human life, cancer accounts for over 10 million new confirmed cases globally each year (Bray et al., 2024). In the realm of clinical treatment, CT is recognized as one of the three traditional therapies, alongside surgery and radiotherapy (Zhu et al., 2024; Wang et al., 2025a). However, the systemic toxic side effects resulting from its non-specific drug distribution remain a significant challenge (Chiang et al., 2024; Chen et al., 2024). Furthermore, it is crucial to note that a single treatment modality often exhibits limited efficacy due to tumor heterogeneity (Li et al., 2025a; Bai et al., 2022). This situation has prompted the medical community to urgently develop a multimodal collaborative treatment system aimed at enhancing treatment effectiveness while minimizing toxicity.

In recent years, tumor treatment strategies based on reactive oxygen species (ROS) have demonstrated revolutionary potential (Chen et al., 2023; Liao et al., 2022; Lee et al., 2022). Among these, CDT has garnered significant attention due to its unique response characteristics to the tumor microenvironment, specifically its ability to catalyze excess H_2_O_2_ in tumor cells to generate highly cytotoxic hydroxyl radicals (•OH) (Zhang et al., 2023a; Liu et al., 2025; Manivasagan et al., 2022; Yan et al., 2025). This endogenous activation mechanism not only circumvents the limitations of light penetration depth associated with photodynamic therapy (PDT) and the dark toxicity issues related to sonodynamic therapy (SDT), but also enables precise tumor-specific killing (Wang et al., 2021). Consequently, the innovative strategy of integrating CDT and CT offers new perspectives for overcoming the limitations of monotherapy and establishing a synergistic anti-cancer system (Li et al., 2024).

The rapid development of nano-drug carrier technology has created an ideal platform for combined CT and CDT (Yu et al., 2023; He et al., 2024; Hou et al., 2023). By functionally designing and integrating nanoparticles, the systemic toxicity of chemotherapy drugs and CDT agents can be effectively reduced while enhancing bioavailability (Julappagari et al., 2025; Li et al., 2025b; Jha et al., 2025; Sun et al., 2023). Researchers have developed a variety of nanoparticles that integrate CT with CDT. For instance, Peng et al. reported a CT/CDT nanoparticle, which successfully targets triple-negative breast cancer by inducing ROS outbreaks and activating immune responses (Wang et al., 2024). However, the clinical therapeutic efficacy of many CT/CDT nanoparticles has not met expectations. To our knowledge, most current CT/CDT nanoparticles exhibit certain limitations, such as a lack of active targeting capabilities and attenuation of CDT efficacy due to the depletion of the H_2_O_2_ substrate (Zhang et al., 2023b; Wang et al., 2019). Furthermore, tumor cells are heterogeneous in various aspects. For example, cancer cells may exist in reducing environments due to elevated levels of intracellular reducing agents, such as glutathione (GSH), which can be several times or even dozens of times higher than those in normal cells. Additionally, many cancer cells have been reported to overproduce ROS. These cells can be found in different tumors and may also coexist in various regions of the same tumor. Moreover, tumor cells at different stages may exhibit varying levels of GSH and ROS (Wang et al., 2013). Consequently, CT/CDT nanoparticles that respond solely to a single type of signal release drugs only in tumor cells that overexpress specific signals, rather than in other tumor cells, resulting in lower overall therapeutic efficacy.

**Fig. 1:**
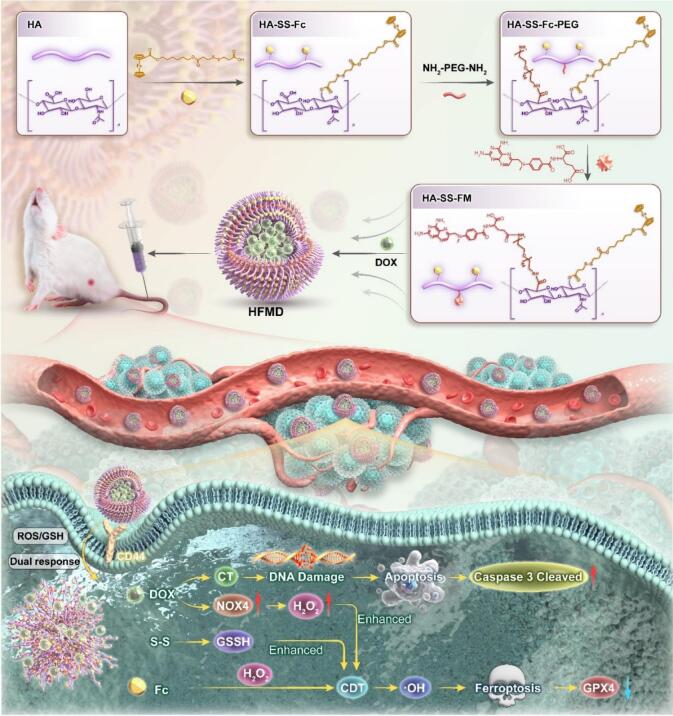
Structural composition and anti-tumor mechanism of HFMD.

In response to the aforementioned scientific challenges, this study developed a redox heterogeneity-responsive CT/CDT nanoparticles, referred to as HFMD (Fig. 1). HFMD integrate multiple functional modules through molecular engineering strategies: hyaluronic acid (HA) acts as a CD44 receptor-targeting ‘navigator’ to facilitate active targeting (Jeannot et al., 2018; Coninx et al., 2022); ferrocene carboxylic acid (Fc) functions both as a Fenton catalyst and a ROS response switch (Na et al., 2021); polyethylene glycol (PEG) prolongs systemic circulation time (Liu et al., 2023); methotrexate (MTX) serves as a hydrophobic core carrier (Yu et al., 2020); and doxorubicin (DOX) not only induces DNA damage by inhibiting topoisomerase II but also upregulates NOX4 expression to enhance H_2_O_2_ supply, thereby forming a self-enhanced CDT cycle (Wang et al., 2025b; Xu et al., 2022; Poursani et al., 2024). The intelligent response mechanism of HFMD was demonstrated through: 1) HA-mediated CD44 targeting, which achieves tumor-specific accumulation; 2) GSH-triggered disulfide bond rupture, leading to controlled drug release; 3) ROS-responsive hydrophobic conversion, ensuring precise drug release; and 4) Fc-catalyzed continuous Fenton reaction coupled with DOX-mediated H_2_O_2_ upregulation, creating a positive feedback loop. This multi-cascade response design effectively addresses the challenge faced by traditional CT/CDT nanoparticles while achieving broad-spectrum adaptability to the heterogeneous tumor microenvironment.

## 2 Materials and methods

### 2.1 Chemicals and Materials

HA (Mw = 52 kDa) was purchased from Bloomage Biotechnology Co., LTD, China. Shanghai aladdin Biochemical Co., Ltd. (China) provided FC, DOX·HCl, 1,6-Hexanediol, Polyethylene glycol diamine (NH_2_-PEG-NH_2_, Mw = 1000 Da), Acetyl chloride, 3, 3’-Dithiodipropionic acid, MTX, 1-ethyl-3(3-dimethyl propylamine) carbodiimide (EDCI), N-Hydroxy succinimide (NHS), fluorescent dye DIR, Tris(2-carboxyethyl) phosphine (TCEP), Ferrostatin-1 (Fer-1) and fluorescent dye BODIPY 581/591 C11. Merck KgaA (Germany) provided FeRhoNox-1 assay kit. Beyotime Biotechnology Co., LTD (China) provided CCK assay kits. Antibodies for CD44, GPX4 and NOX4 were purchased from Proteintech, Hubei (China).

### 2.2 Cell lines and animals

The mouse breast cancer cells (4 T1 cells), Human non-small cell lung cancer cells (A549 cells), Rat normal kidney fibroblasts (NRK-49F cells) were obtained from Procell Life Science&Technology Co., LTD, China. Human umbilical vein endothelial cells (HUVECS cells) were purchased from Haixing Biosciences Co., LTD, China. Mouse ovarian epithelial carcinoma cells (ID8 cells) were obtained from iCell Bioscience Inc., China. Female Balb/c mice (21–22 g) and female SD rats (200 g) were purchased from Liaoning Changsheng Biotechnology Co., LTD, China. All animal experiments in this study were performed in compliance with the ARRIVE guidelines and the Guide for the Care and Use of Laboratory Animals of the National Institutes of Health.

### 2.3 Statistical analysis

The data is expressed as the mean ± standard deviation (SD). To identify differences across several independent groups, a one-way ANOVA was employed. An unpaired *t*-test was used to assess the differences between two groups. Statistically significant differences were observed at both *p* < 0.05 and *p* < 0.01.

Remaining the methodologies were described in the supplementary information.

## 3 Results and discussion

### 3.1 Preparation and properties of HFMD

**Fig. 2:**
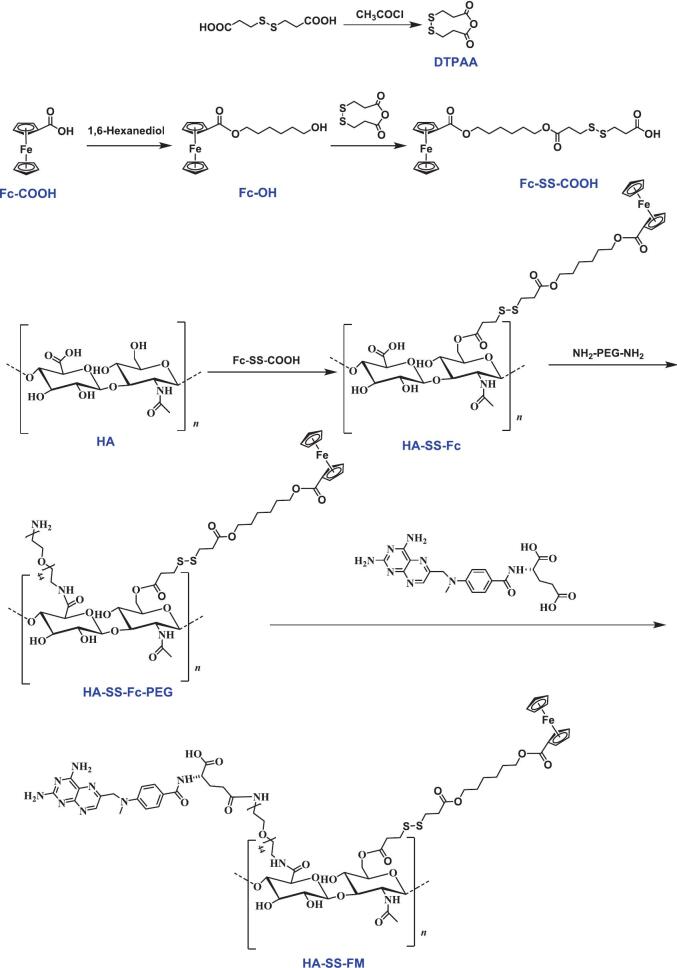
Synthetic route of HA-SS-FM.

**Fig. 3:**
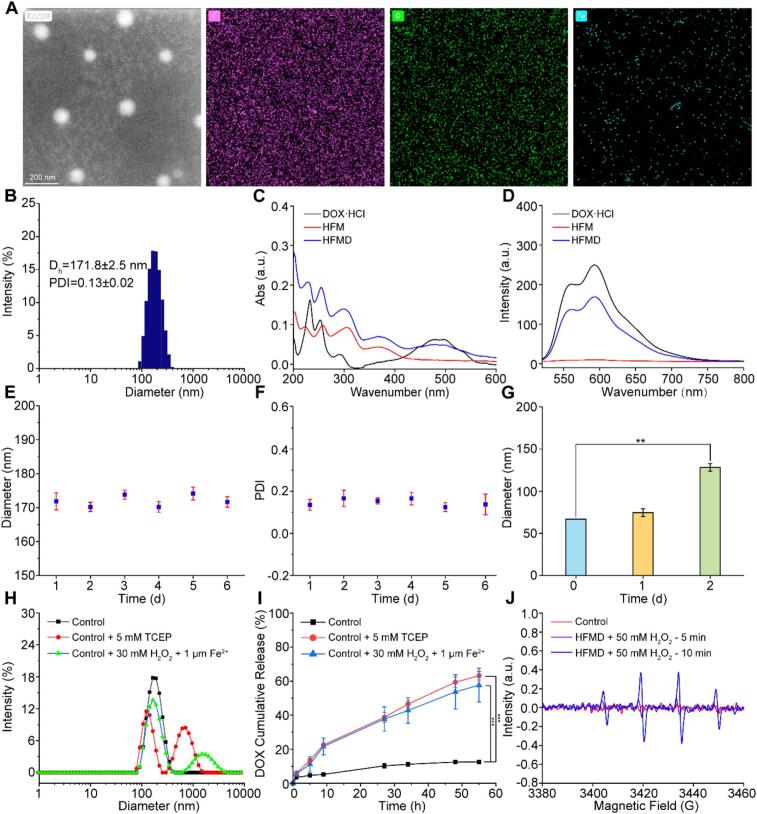
(A) TEM and mapping diagram of HFMD (B) The particle size distribution of HFMD. (C) UV/Vis and (D) fluorescence absorption spectra of DOX·HCl, HFM and HFMD. Particle size (E) and PDI (F) of HFMD within 6 days. (G) Particle size of HFMD in 10 % FBS environment. (H) Particle size distribution curve of HFMD under different conditions. (I) Cumulative drug release curve of DOX·HCl from HFMD. (J) ESR spectra of HFMD. **p < 0.01, ***p < 0.001.

We successfully synthesized the amphiphilic molecule HA-SS-FM by coupling molecules such as HA (H), Fc (F), and MTX (M) (Fig. 2 and Fig. S1). The HA-SS-FM self-assembled and successfully loaded DOX (D) to prepare the nanoparticles HFMD. Transmission electron microscopy, combined with element mapping analysis, revealed that the resulting HFMD exhibited a spherical structure with a regular morphology, and characteristic elements such as carbon (C), oxygen (O), and iron (Fe) displayed a uniform spatial distribution (Fig. 3A). Dynamic light scattering measurements indicated that the hydrated particle size was (171.8 ± 2.5) nm, with a polydispersity index maintained at 0.13 ± 0.02, confirming the system's excellent monodispersity (Fig. 3B). UV–visible absorption spectroscopy and fluorescence spectroscopy analyses demonstrated that the characteristic signal of DOX·HCl was present in HFMD, indicating successful preparation of the formulation (Fig. 3C-D). Stability assessments showed that the particle size distribution of HFMD remained stable over a six-day storage period (Fig. 3E-F); after incubation in a simulated physiological environment containing 10 % fetal bovine serum for 24 h, the system retained its initial particle size characteristics, demonstrating excellent adaptability to biological environments (Fig. 3G and Fig. S2). It is noteworthy that when exposed to high concentrations of GSH and ROS in environments that mimic tumor tissue, HFMD exhibits pronounced structural dissociation and a significantly larger particle size distribution (Fig. 3H and Fig. S3). This observation confirms its redox heterologous response characteristics.

HFMD responds to release under conditions where GSH and ROS are overexpressed, which forms the basis of HFMD's anti-tumor properties. Therefore, we examined the cumulative release rate of DOX·HCl from HFMD under varying conditions. We employed an ultraviolet-visible (UV–Vis) spectrophotometer for testing, which revealed that the encapsulation efficiency and drug loading of HFMD were 72.1 % and 12.6 %, respectively. *In vitro* drug release experiments further corroborate that under conditions of elevated GSH and ROS, which simulate the tumor microenvironment, the cumulative release of DOX·HCl over 55 h reached approximately four times that observed under physiological conditions (Fig. 3I), thereby demonstrating significant stimulus-responsive drug release characteristics.

Additionally, given the presence of ferrocene derivatives in HFMD, we employed electron spin resonance (ESR) technology to assess the performance of HFMD in generating •OH. As illustrated in Fig. 3J, in the presence of H_2_O_2_, the HFMD system reveals notable characteristic peaks of •OH, confirming its ability to convert the overexpressed H_2_O_2_ in tumor cells into highly cytotoxic •OH *via* the Fenton reaction, thereby facilitating CDT treatment. The synergistic effect of this characteristic and the redox-responsive drug release mechanism provides dual assurances for enhancing the efficacy of tumor treatment.

### 3.2 The ability of HFMD to actively target and generate ROS *in vitro*

**Fig. 4:**
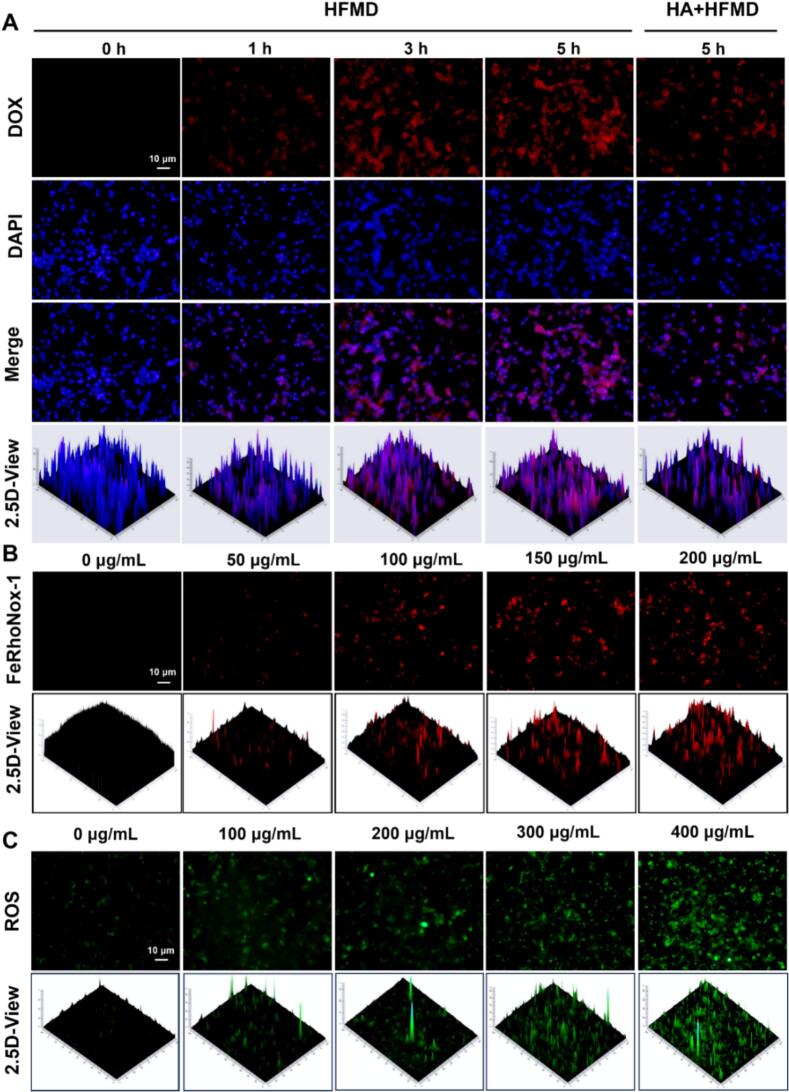
(A) Fluorescence images of HFMD and HA + HFMD taken up by 4 T1 cells. Fluorescence images of (B) Fe^2+^ and (C) ROS levels in 4 T1 cells after incubation with different concentrations of HFM.

The key to achieving accurate drug delivery lies in the active tumor-targeting ability of HFMD. This study evaluated the targeting characteristics of the HFMD using an *in vitro* experimental. Based on the specific binding mechanism of HA to the overexpressed CD44 receptors on the surface of tumor cells, we first employed Western blot analysis to detect the expression levels of CD44 in 4 T1 cells. As illustrated in Fig. S4, 4 T1 cells exhibited significantly elevated CD44 expression compared to normal cells, thereby providing a solid foundation for subsequent research on targeted delivery. Observations from time-gradient experiments revealed that, with the extension of co-incubation time, the red fluorescence intensity in 4 T1 cells increased in a time-dependent manner, indicating that HFMD possesses efficient internalization capabilities (Fig. 4A). Notably, in the HA pre-blocking experiment (HA + HFMD group), the fluorescence signal was significantly reduced compared to the 5-h HFMD group. The flow cytometry results were consistent with the above results (Fig. S5). In addition, we observed that normal NRK-49F cells with low expression of CD44 uptake HFMD, and after 5 h, the intracellular fluorescence intensity was significantly lower than that of 4 T1 cells (Fig. S6). The phenomenon strongly confirms that HFMD can effectively target 4 T1 cells and achieve targeted drug delivery.

**Fig. 5:**
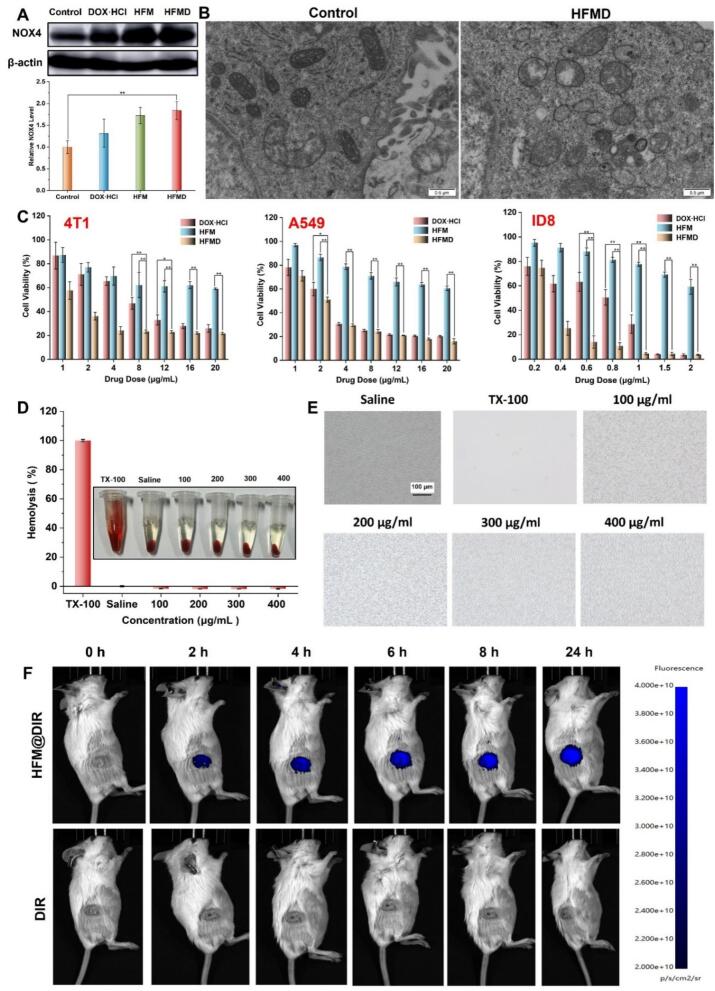
(A) NOX4 expression levels of cells after incubation with different preparations. (B) Bio-TEM images of 4 T1 cells after HFMD. (C) The cytotoxic effects of HFMD were evaluated on 4 T1, A549, and ID8 cells. (D) Hemolysis rate and (E) red blood cell pictures of HA-SS-FM. (F) Fluorescence imaging of mice after tail vein injection of HFM@DIR. **p < 0.01. (For interpretation of the references to colour in this figure legend, the reader is referred to the web version of this article.)

HFMD can be effectively taken up by tumor cells, which inspired us to utilize the FerhoNox-1 kit to assess iron levels in 4 T1 cells following exposure to HFMD. Given that the fluorescence spectra of DOX overlap with those of the FerhoNox-1 probe, and considering that changes in divalent iron ions levels are indicated by HFM, we incubated 4 T1 cells with varying concentrations of HFM for 5 h before measuring the intracellular levels of divalent iron ions. As illustrated in the Fig. 4B, the red fluorescence intensity in cells gradually increases with the rising concentration of HFM. This finding demonstrates that HFMD can elevate the concentration of divalent iron ions within cells, thereby achieving CDT and inhibiting tumor growth. To further investigate the capacity of HFMD to induce the upregulation of ROS in cells, we employed the DCFH-DA kit. The results, depicted in the Fig. 4C, show that the intracellular green fluorescence intensity increases progressively with higher HFM concentrations, confirming that HFMD induces the upregulation of ROS in tumor cells. A significant limitation of most current CT/CDT nanoparticles is the attenuation of CDT due to H_2_O_2_ substrate depletion. Numerous studies have reported that DOX can upregulate NOX4 expression and enhance H_2_O_2_ supply, thereby creating a self-reinforcing CDT cycle. In light of this, we utilized WB technology to investigate whether HFMD can upregulate NOX4 protein expression. As shown in the Fig. 5A, the protein expression of NOX4 in the HFMD group was significantly upregulated compared to the blank group, demonstrating that HFMD enhances H_2_O_2_ supply and improves CDT efficiency. HFMD increases the intracellular concentration of divalent iron ions, leading to a substantial rise in ROS. These characteristics align with the phenomenon of ferroptosis, an iron-dependent form of regulated cell death resulting from excessive accumulation of lipid peroxides (Mao et al., 2021). Numerous studies have reported that promoting ferroptosis in tumor cells can inhibit tumor growth and enhance anti-cancer efficacy (Bae et al., 2022; Guo et al., 2024). A typical characteristic of ferroptosis is the downregulation of GPX4 and the occurrence of lipid peroxidation (Yuan et al., 2023; Chen et al., 2025). Therefore, we employed Western Blot techniques along with the fluorescent dye BODIPY 581/591 C11 and biological electron microscopy to further investigate whether HFMD can induce ferroptosis in tumor cells. As shown in Fig. S7, HFMD treatment significantly reduced GPX4 protein levels. To further validate its correlation with ferroptosis, we administered the ferroptosis inhibitor Fer-1 alongside HFMD treatment to the 4 T1 cells, and assessed GPX4 expression after 24 h. The results indicated that, compared to the use of HFMD alone, the combined treatment of HFMD and Fer-1 significantly upregulated GPX4 protein expression (Fig. S8). In addition, we attempted to evaluate whether HFMD induces lipid peroxidation using the C11-BODIPY probe. However, the experiment was unsuccessful due to the spectral overlap between the DOX contained in HFMD and the C11-BODIPY probe. Considering that the lipid peroxidation induced by HFMD is primarily attributed to its Fc component, we shifted our approach to co-incubate HFM, which does not contain DOX, with tumor cells for 5 h, followed by C11-BODIPY staining. The results showed that the ratio of green to red fluorescence signals in the HFM-treated group was significantly higher than that in the control group, indicating that HFM indeed caused significant lipid peroxidation (Fig. S9). Results from biological electron microscopy revealed that, in comparison to the blank group, the mitochondria of 4 T1 cells in the HFMD group exhibited shrinkage and loss of cristae structure (Fig. 5B). In summary, the above experimental results collectively demonstrate that HFMD can effectively induce ferroptosis in tumor cells by downregulating GPX4 protein expression and promoting lipid peroxidation.

### 3.3 Cytotoxicity and hemocompatibility

In this study, the CCK8 method was employed to systematically evaluate the cytotoxic effects of HFMD on three cancer cell lines: 4 T1, A549, and ID8. As illustrated in Fig. 5C, an increase in drug concentrations resulted in a significant dose-dependent decline in the survival rates of these three cancer cell lines. This finding robustly supports the assertion that HFMD possesses notable anti-tumor activity. From a clinical translation perspective, it is essential for an ideal nano-delivery system for cancer treatment to exhibit good blood compatibility. In light of this research requirement, we conducted an experimental evaluation of the hemolysis of HA-SS-FM. The experimental results, presented in Fig. 5D, indicate that within the concentration range of 400 μg/mL, the hemolysis rate of HA-SS-FM remained below the internationally recognized safety threshold of 5 % (Luo et al., 2022). Furthermore, observations from an inverted fluorescence microscope revealed that the red blood cells retained their normal morphology (Fig. 5E). These findings suggest that HA-SS-FM demonstrates excellent hemocompatibility and fully complies with safety requirements for intravenous administration.

### 3.4 Antitumor activity of HFMD

**Fig. 6:**
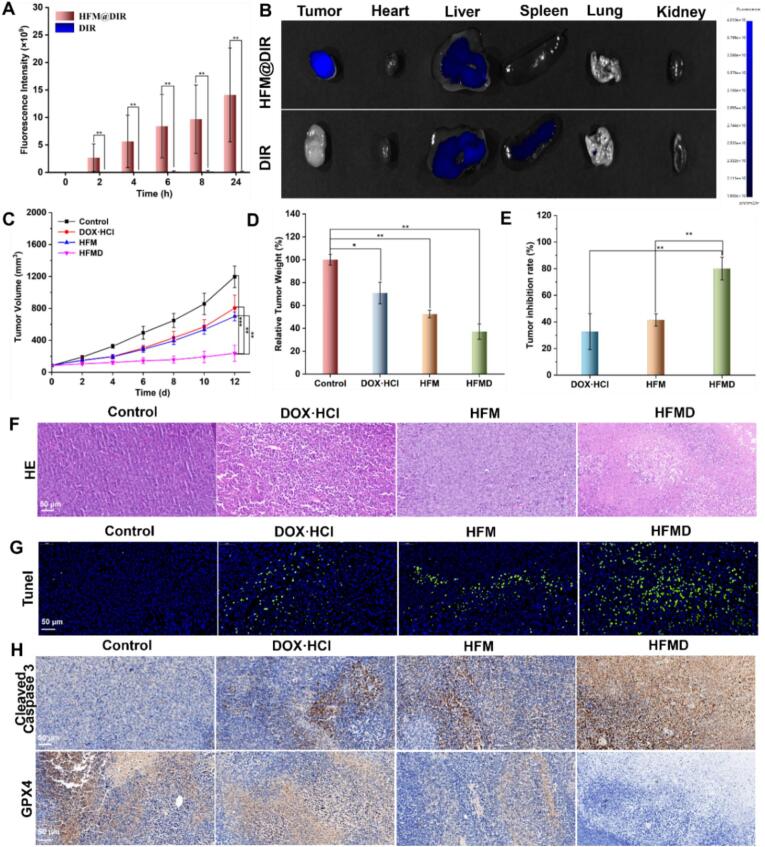
(A) Quantitative diagram of Fig. 5F. (B) Fluorescence imaging of mice organs and tumors after 24 h of tail vein injection of HFM@DIR. Change curve of tumor volume (C) of mice during treatment. Tumor weight (D) and tumor inhibition rate (E) of mice in each group. Tumor analysis by (F) HE, (G) TUNEL staining, and (H) immunocytochemistry across treatment groups. *p < 0.05, **p < 0.01, ***p < 0.001.

Given the excellent *in vitro* physical and chemical properties and significant antitumor activity of HFMD, this study further established a 4 T1 tumor-bearing mouse model to evaluate its antitumor effect *in vivo*. When nanoparticles are injected into the body, a good blood drug concentration is fundamental for outstanding nanoparticles; therefore, we examined the blood drug concentration of DOX in HFMD after tail vein injection. As shown in Fig. S10, HFMD exhibited higher drug concentrations in the blood compared to free DOX·HCl, which may be attributed to the appropriate nanoscale of HFMD and the PEG chains on its surface. To achieve efficient tumor treatment, nanoparticles must possess tumor-targeted enrichment capabilities. To test this hypothesis, we employed a small animal *in vivo* imaging system to dynamically monitor nanoparticles accumulation in tumors. Considering the tissue permeability limitations of DOX, we utilized the near-infrared fluorescent probe DIR, which has deep penetration capabilities, to construct the nanoparticles HFM@DIR. *In vivo* imaging results indicate that, compared to the free DIR group, the HFM@DIR group exhibited significantly enhanced fluorescence signals in the tumor regions at all time points post-injection. The fluorescence signal intensity of tumors in the HFM@DIR group at 24 h was approximately 39.3 times that of the DIR group (Fig. 5F, Fig. 6A-B and Fig. S11), thereby confirming that HFMD possesses excellent tumor-targeting enrichment capability. Subsequently, we administered DOX·HCl and HFMD *via* tail vein injection into tumor-bearing mice, measuring the DOX content in various organs and tumors 24 h later. As illustrated in Fig. S12, the DOX content in the tumors of the HFMD group was significantly higher than that of the DOX·HCl group, further demonstrating the excellent active targeting capability of HFMD towards tumor with high CD44 expression. *In vivo* anti-tumor experimental data demonstrated that after 12 days of treatment, the tumor volume and weight of mice in the HFMD treatment group were significantly lower than those in the control groups, with a tumor inhibition rate reaching 80.1 % (Fig. 6C-E and Fig. S13). This finding robustly supports the excellent anti-cancer efficacy of HFMD. Histopathological analysis, *via* H&E staining, revealed that tumor cells in the control group exhibited characteristic features of malignant proliferation, whereas the HFMD group displayed extensive disintegration of cellular structures (Fig. 6F). Tunel assay results indicated that the intensity of the green fluorescent signal in the HFMD group was significantly higher than in the control group, confirming that HFMD induces apoptosis in tumor cells (Fig. 6G). Furthermore, immunohistochemical analysis revealed an up-regulation of Cleaved caspase 3 levels in the tumor tissues of the HFMD group, aligning with the apoptosis detection results (Fig. 6H). Concurrently, the expression level of the GPX4 protein was found to be decreased, thereby confirming the effective activation of the ferroptosis pathway (Fig. 6H). Collectively, these multidimensional evidences indicate that HFMD exerts a combined anti-tumor effect through the synergistic activation of the caspase-dependent apoptosis pathway and the GPX4-regulated ferroptosis mechanism.

### 3.5 Biosafety of HFMD

**Fig. 7:**
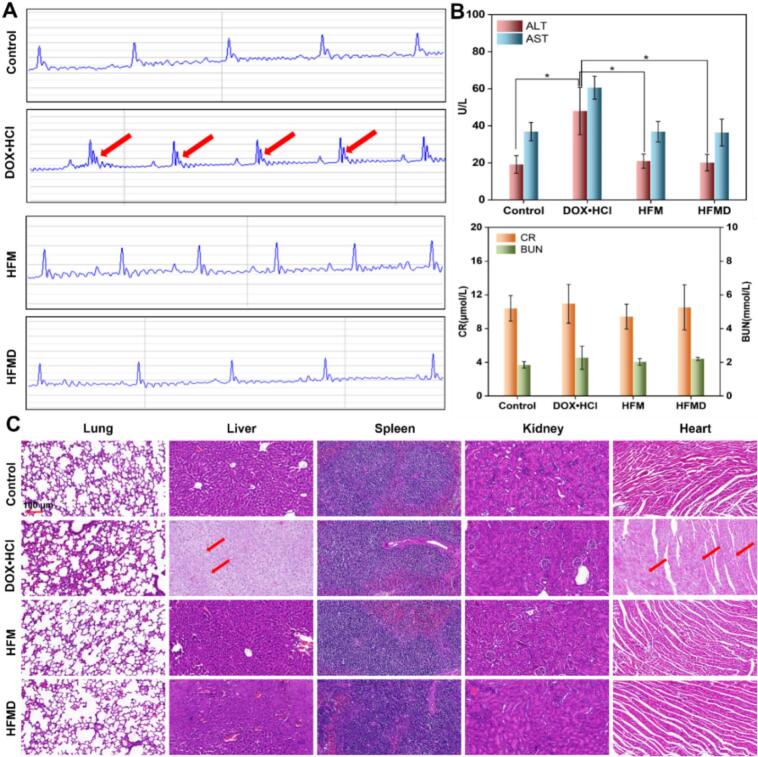
(A) Electrocardiograms of mice in each group after treatment. Post-treatment analysis included serum (B) ALT/AST, CR/BUN levels, and (C) histological examination of major organs in all groups. *p < 0.05.

To ensure the safety of nanomaterials in clinical applications, we systematically evaluated the *in vivo* compatibility of HFMD. The change in body mass of tumor-bearing mice, recorded every other day, served as a key indicator of systemic toxicity and was monitored throughout the experiment. The data indicated that the body weight growth curves of both the HFMD group and the blank control group were highly consistent (Fig. S14). A multi-dimensional safety assessment of treatment endpoints revealed no statistically significant differences in blood biochemical indicators (ALT, AST, CR, BUN), Electrocardiograms parameters, and histopathological examination results of major organs, thereby confirming the biological safety of the preparation (Fig. 7A-C). Notably, the DOX·HCl treatment group exhibited a significant decrease in body mass, accompanied by characteristic myocardial damage, which manifested as pathological changes such as abnormal elevation of the ST segment in the electrocardiogram. This dose-dependent toxic reaction is highly consistent with the clinically observed cardiotoxicity characteristics of anthracyclines. Furthermore, the livers of mice in the DOX·HCl treatment group exhibited some degree of damage (Fig. 7B-C). To further investigate the biosafety of HFMD, we conducted a long-term toxicity evaluation over a period of 25 days. The results indicated that, after 25 days, the HE staining indices and biochemical analysis indicators of various organs in HFMD mice showed no significant differences compared to the control group (Fig. S15). This study confirms that HFMD effectively mitigate the toxicity associated with free DOX·HCl while preserving anti-tumor efficacy through their unique drug delivery mechanism. Notably, they significantly reduce the risk of cardiac and liver toxicity, demonstrating promising prospects for clinical translation.

## 4 Conclusion

This study successfully developed a CT/CDT nanoparticle system for HFMD based on a redox heterogeneity response mechanism. *In vitro* experiments and evaluations using animal models demonstrated that the HFMD exhibits significant anticancer effects. HFMD possesses several remarkable characteristics: first, the optimized nanoparticle size (171.8 ± 2.5 nm) and uniform morphology facilitate the enhanced permeability and retention effect for passive targeting of tumor tissues; second, HFMD can achieve active targeting by binding to CD44 receptors on the surface of tumor cells; third, its unique redox response properties can trigger drug release in the tumor microenvironment and enhance the CDT effect by upregulating intracellular H_2_O_2_ levels. Mechanistic studies have shown that HFMD effectively induces tumor cell death through a synergistic mechanism involving ferroptosis and apoptosis. In a tumor-bearing mouse model, the tumor inhibition rate reached 80.1 %. Importantly, systematic toxicity assessments confirmed that HFMD demonstrates good biocompatibility and holds promise for clinical translation.

## CRediT authorship contribution statement

**Su Cui:** Writing – original draft. **Li Yu:** Writing – original draft. **Hao Liu:** Writing – original draft. **Wenhan Liu:** Writing – review & editing. **Daiwang Shi:** Writing – review & editing.

## Declaration of competing interest

The authors declare that they have no known competing financial interests or personal relationships that could have appeared to influence the work reported in this paper.

## Data Availability

Data will be made available on request.

## Appendix A Supplementary data

Supplementary material Supporting information：Tumor Redox Heterogeneity-Responsive Nanoparticles for Enhanced Antitumor Efficacy through Combining Chemo/Chemodynamic Therapy. Supplementary material

## References

[bb0005] Bae C., Kim H., Kook Y., Lee C., Kim C., Yang C., Park M.H., Piao Y., Koh W., Lee K. (2022). Induction of ferroptosis using functionalized iron-based nanoparticles for anti-cancer therapy. Mater. Today Bio.

[bb0010] Bai Y., Zhao J., Zhang L., Wang S., Hua J., Zhao S., Liang H. (2022). A smart near-infrared carbon dot-metal organic framework assemblies for tumor microenvironment-activated cancer imaging and chemodynamic-photothermal combined therapy. Adv. Healthc. Mater..

[bb0015] Bray F., Laversanne M., Sung H., Ferlay J., Siegel R.L., Soerjomataram I., Jemal A. (2024). Global cancer statistics 2022: globocan estimates of incidence and mortality worldwide for 36 cancers in 185 countries. CA Cancer J. Clin..

[bb0020] Chen J., Zhan Q., Li L., Xi S., Cai L., Liu R., Chen L. (2025). Cell-membrane targeting sonodynamic therapy combination with fsp1 inhibition for ferroptosis-boosted immunotherapy. Mater. Today Bio.

[bb0025] Chen Y., Yang Z., Wang S., Ma Q., Li L., Wu X., Guo Q., Tao L., Shen X. (2023). Boosting ros-mediated lysosomal membrane permeabilization for cancer ferroptosis therapy. Adv. Healthc. Mater..

[bb0030] Chen Y., Lu Z., Wang D. (2024). Multifunctional nanoplatform for single nir laser-regulated efficient pdt/ptt/chemotherapy. Biomacromolecules.

[bb0035] Chiang S.T., Chen Q., Han T., Qian C., Shen X., Lin Y., Xu R., Cao Z., Zhou C., Lu H., Li R., Ai X. (2024). Biomimetic nanovesicles synergize with short-term fasting for enhanced chemotherapy of triple-negative breast cancer. ACS Nano.

[bb0040] Coninx S., Kalot G., Godard A., Bodio E., Goze C., Sancey L., Auzély-Velty R. (2022). Tailored hyaluronic acid-based nanogels as theranostic boron delivery systems for boron neutron cancer therapy. Intern. J. Pharmac.: X.

[bb0045] Guo Y., Luo H., Jiang H., Liu X., Long X., Hou Y., Chen Z., Sun Y., Ge D., Shi W. (2024). Liposome encapsulated polydopamine nanoparticles: enhancing ferroptosis and activating hypoxia prodrug activity. Mater. Today Bio.

[bb0050] He X., Li M., Fan S., Li Y., Fang L., Xiang G., Yang T. (2024). Copper peroxide and cisplatin co-loaded silica nanoparticles-based trinity strategy for cooperative cuproptosis/chemo/chemodynamic cancer therapy. Chem. Eng. J..

[bb0055] Hou R., Lu T., Sun J., Li D., Xu L., Zhang R., Yu Z. (2023). Biodegradable nanotherapeutic with simultaneously gsh depletion and h2o2 supplying for enhanced synergistic chemotherapy/chemodynamic therapy. Mater. Des..

[bb0060] Jeannot V., Gauche C., Mazzaferro S., Couvet M., Vanwonterghem L., Henry M., Didier C., Vollaire J., Josserand V., Coll J., Schatz C., Lecommandoux S., Hurbin A. (2018). Anti-tumor efficacy of hyaluronan-based nanoparticles for the co-delivery of drugs in lung cancer. J. Control. Release.

[bb0065] Jha D., Kumar P., Gautam H.K. (2025). Citrus maxima extract-coated versatile gold nanoparticles display ros-mediated inhibition of mdr-pseudomonas aeruginosa and cancer cells. Bioorg. Chem..

[bb0070] Julappagari M., Haque S., Tripathy S., Londhe S., Patel A., Banerjee R., Patra C.R. (2025). Gold nanoparticles-based targeted delivery of rapamycin and olaparib to breast cancer: an in vitro and in vivo approach. Bioorg. Chem..

[bb0075] Lee J., Davaa E., Jiang Y., Shin K., Kim M.H., An H., Kim J., Cho S.K., Yang S. (2022). Pheophorbide a and sn38 conjugated hyaluronan nanoparticles for photodynamic- and cascadic chemotherapy of cancer stem-like ovarian cancer. Carbohydr. Polym..

[bb0080] Li J., Chen Z., Pei Z., Pei Y. (2025). A hyaluronic acid modified advanced nanoagent activated by tumor microenvironment enables a reciprocal dual-modality therapy. Chem. Eng. J..

[bb0085] Li K., Gui S., Wang N., Li X., Zhao C., Liu M., Zhang Z. (2025). Sequential ph/gsh-responsive stealth nanoparticles for co-delivery of anti-pd-1 antibody and paclitaxel to enhance chemoimmunotherapy of lung cancer. Eur. J. Med. Chem..

[bb0090] Li Z., Chen S., Zhu J. (2024). Dual noncovalent binding modes enabled via single thiourea motif: an emerging arsenal for supramolecular polymeric nanomedicine. Adv. Funct. Mater..

[bb0095] Liao J., Huang Q., Li Y., Zhang D., Wang G. (2022). Chitosan derivatives functionalized dual ros-responsive nanocarriers to enhance synergistic oxidation-chemotherapy. Carbohydr. Polym..

[bb0100] Liu M., Liu S., Xiu J., Zhang J., Deng Y., Zhao X. (2023). “Y-type” peg modified liposomes could eliminate the accelerated blood clearance (abc) phenomenon and improved tumor therapy. Appl. Mater. Today.

[bb0105] Liu Y., Zhang X., Zhang X., Wang G., Li X., Xing S., Cao C., Li Y., Han L., Wang S. (2025). Histone deacetylase inhibiting nanoprodrugs for enhanced chemodynamic therapy through multistage downregulating glutathione. Int. J. Biol. Macromol..

[bb0110] Luo J., Ma Z., Yang F., Wu T., Wen S., Zhang J., Huang L., Deng S., Tan S. (2022). Fabrication of laponite-reinforced dextran-based hydrogels for nir-responsive controlled drug release. ACS Biomater Sci. Eng..

[bb0115] Manivasagan P., Joe A., Han H., Thambi T., Selvaraj M., Chidambaram K., Kim J., Jang E. (2022). Recent advances in multifunctional nanomaterials for photothermal-enhanced Fenton-based chemodynamic tumor therapy. Mater. Today Bio.

[bb0120] Mao C., Liu X., Zhang Y., Lei G., Yan Y., Lee H., Koppula P., Wu S., Zhuang L., Fang B., Poyurovsky M.V., Olszewski K., Gan B. (2021). Dhodh-mediated ferroptosis defence is a targetable vulnerability in cancer. Nature.

[bb0125] Na Y., Woo J., Choi W.I., Sung D. (2021). Novel carboxylated ferrocene polymer nanocapsule with high reactive oxygen species sensitivity and on-demand drug release for effective cancer therapy. Colloids Surf. B: Biointerfaces.

[bb0130] Poursani E., Cirillo G., Curcio M., Vittorio O., De Luca M., Leggio A., Nicoletta F.P., Iemma F. (2024). Dual-responsive chondroitin sulfate self-assembling nanoparticles for combination therapy in metastatic cancer cells. Intern. J. Pharmac.: X.

[bb0135] Sun L., Liu H., Ye Y., Lei Y., Islam R., Tan S., Tong R., Miao Y.B., Cai L. (2023). Smart nanoparticles for cancer therapy. Signal Transduct. Target. Ther..

[bb0140] Wang J., Sun X., Mao W., Sun W., Tang J., Sui M., Shen Y., Gu Z. (2013). Tumor redox heterogeneity-responsive prodrug nanocapsules for cancer chemotherapy. Adv. Mater..

[bb0145] Wang N., Liu C., Yao W., Zhou H., Yu S., Chen H., Qiao W. (2021). A traceable, sequential multistage-targeting nanoparticles combining chemo/chemodynamic therapy for enhancing antitumor efficacy. Adv. Funct. Mater..

[bb0150] Wang N., Liu Y., Peng D., Zhang Q., Zhang Z., Xu L., Yin L., Zhao X., Lu Z., Peng J. (2024). Copper-based composites nanoparticles improve triple-negative breast cancer treatment with induction of apoptosis-cuproptosis and immune activation. Adv. Healthc. Mater..

[bb0155] Wang Q., Cheng S., Han C., Yang S., Gao S., Yin W., Song W. (2025). Tailoring cell-inspired biomaterials to fuel cancer therapy. Mater. Today Bio.

[bb0160] Wang S., Wang Z., Yu G., Zhou Z., Jacobson O., Liu Y., Ma Y., Zhang F., Chen Z.Y., Chen X. (2019). Tumor-specific drug release and reactive oxygen species generation for cancer chemo/chemodynamic combination therapy. Adv. Sci..

[bb0165] Wang T., Li Z., Lei J., Zhang Y., Tong Y., Guan X., Wang S. (2025). Rgd peptide-functionalized micelles loaded with crocetin ameliorate doxorubicin-induced cardiotoxicity. Intern. J. Pharmac.: X.

[bb0170] Xu W., Wang T., Qian J., Wang J., Hou G., Wang Y., Cui X., Suo A., Wu D. (2022). Fe(ii)-hydrazide coordinated all-active metal organic framework for photothermally enhanced tumor penetration and ferroptosis-apoptosis synergistic therapy. Chem. Eng. J..

[bb0175] Yan X., Liu H., Guo L., Liu C., Zhang S., Wang X., Tang Y., Zhou R., Jiang X., Wang E., Gao S., Xu C. (2025). Multifunctional drug delivery nanoparticles for combined chemotherapy/chemodynamic/photothermal therapy against colorectal cancer through synergistic cuproptosis/ferroptosis/apoptosis. Mater. Today Bio.

[bb0180] Yu F., Zhu M., Li N., Ao M., Li Y., Zhong M., Yuan Q., Chen H., Fan Z., Wang Y., Hou Z., Qi Z., Shen Y., Chen X.D. (2020). Imaging-guided synergistic targeting-promoted photo-chemotherapy against cancers by methotrexate-conjugated hyaluronic acid nanoparticles. Chem. Eng. J..

[bb0185] Yu H., Zhao H., Zhang Y., Hou Y., Li R., Liang T., Zhang Y., Li C., Zhao J., Zhang M., An R. (2023). A biomimetic nanoreactor for combinational chemo/chemodynamic therapy of choriocarcinoma through synergistic apoptosis and ferroptosis strategy. Chem. Eng. J..

[bb0190] Yuan Y., Tian C., Wang Q., Qiu X., Wang Y., Jiang H., Hao J., He Y. (2023). Synergistic amplification of ferroptosis with liposomal oxidation catalyst and gpx4 inhibitor for enhanced cancer therapy. Adv. Healthc. Mater..

[bb0195] Zhang X., Guo H., Zhang X., Shi X., Yu P., Jia S., Cao C., Wang S., Chang J. (2023). Dual-prodrug cascade activation for chemo/chemodynamic mutually beneficial combination cancer therapy. Biomater. Sci..

[bb0200] Zhang Y., Wang F., Shi L., Lu M., Lee K., Ditty M.M., Xing Y., He H., Ren X., Zheng S. (2023). Nanoscale coordination polymers enabling antioxidants inhibition for enhanced chemodynamic therapy. J. Control. Release.

[bb0205] Zhu S., Jin G., He X., Li Y., Xu F., Guo H. (2024). Mechano-assisted strategies to improve cancer chemotherapy. Life Sci..

