# Supplementary material for: Tumor redox heterogeneity-responsive nanoparticles for enhanced antitumor efficacy through combining chemo/chemodynamic therapy
Source: Int J Pharm X. 2025 Nov 24;10:100455. doi: 10.1016/j.ijpx.2025.100455 (PMC12702408; doi:10.1016/j.ijpx.2025.100455)
Supplement: Supplementary material — Supporting information：Tumor Redox Heterogeneity-Responsive Nanoparticles for Enhanced Antitumor Efficacy through Combining Chemo/Chemodynamic Therapy. [file mmc1.docx]

Tumor Redox Heterogeneity-Responsive Nanoparticles for Enhanced Antitumor Efficacy through Combining Chemo/Chemodynamic Therapy

Su Cui^2,#^, Li Yu^3,#^, Hao Liu^4,#^, Wenhan Liu^1*^, Daiwang Shi^1*^

^1^Department of Thoracic Surgery, Shengjing Hospital of China Medical University, Shenyang, 110004, China

^2^Department of Thoracic Surgery, First Affiliated Hospital of China Medical University, Shenyang, 110001, China

^3^Department of Oncology, Shengjing Hospital of China Medical University, Shenyang, 110004, China

^4^Department of Obstetrics and Gynecology, Shengjing Hospital of China Medical University, Shenyang, 110004, China

^#^These authors contribute equally: Su Cui, Li Yu and Hao Liu

*Corresponding author

Dr. Daiwang Shi

Department of Thoracic Surgery, Shengjing Hospital of China Medical University, Shenyang, 110004, China

Email: cmu_daiwang@163.com

**Experimental section**

- **Synthesis of HA-SS-FM**

3,3'-Dithiodipropionic acid (2 g, 9.5 mmol) was added to 10 mL of acetyl chloride and refluxed at 65°C for 2 h. The solvent was removed by rotary evaporation and the resulting solid was repeatedly washed with cold ether and placed in a vacuum drying chamber of 35°C to obtain DTPAA (1.2 g, 65%) as a white solid.

Fc (230 mg, 1 mmol), EDCI (230 mg, 1.2 mmol), DMAP (150 mg, 1.22 mmol) and 1,6-hexanediol (130 mg, 1.1 mmol) was dissolved in 80 mL anhydrous DCM, the reaction was stirred for 24 h at room temperature. Subsequently, the mixture was washed with 0.1 M HCl and water, dried over anhydrous Na_2_SO_4_, and concentrated into the crude product, which was purified by silica gel chromatography to give Fc-OH (261 mg, yield 79%). ^1^H NMR (400 MHz, CDCl_3_): δ/ppm = 4.82 (t, 2H,); 4.41 (t, 2H); 4.22 (m, 7H); 3.69 (t, 2H); 1.76 (m, 2H); 1.62 (m, 2H); 1.49 (m, 4H).

Fc-OH (330 mg, 1 mmol), DMAP (122 mg, 1 mmol) and DTPAA (364 mg, 2 mmol) was dissolved in 100 mL anhydrous DCM, the reaction was stirred for 24 h at room temperature. Next, the mixture was washed with 0.1 M HCl and water, dried over anhydrous Na_2_SO_4_, and concentrated into the crude product, which was purified by silica gel chromatography to give Fc-SS-COOH (397 mg, yield 76.1%). ^1^H NMR (400 MHz, CDCl_3_): δ/ppm = 4.81 (s, 2H,); 4.40 (s, 2H); 4.20 (m, 7H); 4.13 (t, 2H); 2.94 (t, 4H); 2.77(m, 4H); 1.70 (m, 4H); 1.47 (m, 4H).

HA (460 mg), Fc-SS-COOH (521 mg, 1 mmol), EDCI (384 mg, 2 mmol) and DMAP (122 mg, 1 mmol) were dissolved in 100 mL DMSO, and the reaction was stirred for 24 h at 30 °C. Next, the product was purified by dialyzing in a membrane (8 kDa) against deionized for 48 h and filtered lyophilized to obtain the HA-SS-Fc (557 mg). The successful synthesis of HA-SS-Fc was confirmed by ^1^H-NMR.

HA-SS-Fc (500 mg), NH_2_-PEG-NH_2_ (1 g, 1 mmol), EDCI (192 mg, 1 mmol) and NHS (115 mg, 1 mmol) were dissolved in 100 mL DMSO, and the reaction was stirred for 24 h at 30 °C. Subsequently, the product was purified by dialyzing in a membrane (14 kDa) against deionized for 72 h and filtered lyophilized to obtain the HA-SS-Fc-PEG (845 mg). The successful synthesis of HA-SS-Fc-PEG was confirmed by ^1^H-NMR.

HA-SS-Fc-PEG (1000 mg), MTX (454, 1 mmol), EDCI (192 mg, 1 mmol) and NHS (115 mg, 1 mmol) were dissolved in 100 mL DMSO, and the reaction was stirred for 24 h at 30 °C. Subsequently, the product was purified by dialyzing in a membrane (14 kDa) against deionized for 48 h and filtered lyophilized to obtain the HA-SS-FM (1.21 g). The successful synthesis of HA-SS-FM was confirmed by ^1^H-NMR.

- **Preparation and Characteristic Evaluation of HFMD**

To prepare HFMD, 5 mg of HA-SS-FM was dissolved in 5 mL of DOX·HCl solution (0.2 mg/mL) and stirred vigorously overnight at room temperature. The solution was subsequently dialyzed using a dialysis bag with a molecular weight cut-off of 8 kDa for 8 hours. In a separate procedure, 5 mg of HA-SS-FM was dissolved in 5 mL of PBS solution and stirred vigorously overnight to produce the nanoparticle HFM. The particle size and morphology of HFMD were characterized using dynamic light scattering (DLS) and transmission electron microscopy (TEM). The HFMD was placed in a dialysis bag with a molecular weight cut-off of 3500 Da and incubated in 100 mL of pH 7.4 PBS solution. Additionally, to simulate the tumor microenvironment, separate experiments were conducted using pH 7.4 PBS solution with 5 mM TCEP and pH 7.4 PBS solution containing 30 mM H_2_O_2_ and 1 μM Fe^2+^. At specific time intervals, the sample solution was withdrawn and replaced with an equivalent volume of fresh solution. The concentrations of DOX·HCl in the PBS solution were measured using UV-Vis spectroscopy, and the cumulative drug release rate was subsequently calculated.

- **Evaluation of HA-SS-FM blood compatibility**

Blood samples from Balb/c mice were diluted with saline at a ratio of 1:25 and placed into a 24-well plate. Various concentrations of HA-SS-FM solution (100, 200, 300, and 400 μg/mL) were subsequently added and incubated for 4 hours at 37 °C. Following this incubation, 0.9 mL of the blood was transferred to a test tube and centrifuged for 5 minutes at 1500 rpm to eliminate intact red blood cells. Subsequently, 100 μL of the supernatant was pipetted into a 96-well plate for absorbance measurement at 540 nm using a microplate reader. The red blood cells were treated with 1% Triton X-100 and served as a positive control.

- **Cellular uptake**

The 4T1 cells were cultured overnight in a 24-well plate at 37 °C in a 5% CO_2_ environment. Following this incubation, the medium was replaced, and HFMD was introduced to the 4T1 cells for durations of 0, 1, 3, and 5 hours. Subsequently, the medium was removed, and the cells were rinsed with PBS. DAPI staining was performed for 10 minutes, after which the cells were observed using an inverted fluorescence microscope (IFM; ZEISS Axio Observer, Germany).

The 4T1 cells were plated in six-well plates and incubated overnight at 37 °C in a 5% CO_2_ atmosphere. The medium was refreshed, and various concentrations of HFM solution (0, 50, 100, 150, and 200 μg/mL) were then added. After a 5-hour incubation, the cells were washed with PBS and stained with FeRhoNox-1. Following an additional 2 hours incubation, fluorescence images were analyzed using the IFM.

- **HFM produces ROS capability**

The 4T1 cells were seeded in six-well plates and cultured overnight. Subsequently, various concentrations of HFM solution (0, 100, 200, 300, and 400 μg/mL) were added. After a 5-hour incubation, the culture medium was removed, and the cells were rinsed three times with PBS before being stained with DCFH-DA. Following a 30-minute incubation, fluorescence images were captured using an IFM.

- **Cell viability**

The 4T1, A549, and ID8 cell lines were individually seeded in 96-well plates and allowed to culture overnight. Following this, the cells were divided into three treatment groups: DOX·HCl, HFM, and HFMD. Subsequently, the appropriate preparations were introduced according to the designated group. After a 24-hour incubation period, 10 μL of CCK-8 reagent was added to each well and incubated for an additional hour. The absorbance was then measured using a microplate reader set to 450 nm.

- **Western blot analysis**

The 4T1 cells were cultured overnight in 96-well plates. These cells were divided into four groups: Control, DOX·HCl, HFM, and HFMD, with treatments administered according to the group designation. After 24 hours, the cells were harvested, total protein was extracted, and quantification was performed using a BCA protein assay kit. The protein samples (40-50 μg) were separated using PAGE@Gel. Following this, the membranes were incubated with primary antibodies specific to NOX4 and GPX4, followed by treatment with an appropriate horseradish peroxidase-conjugated secondary antibody. The process for testing protein expression of CD44 in NRK-49F cells, HUVCES, and 4T1 cells is analogous to the aforementioned method.

- ***In vivo* anti-cancer efficacy and biosafety evaluation**

4T1 cells (1×10^7^) were combined with a specific quantity of matrix gel and subsequently injected beneath the breast pad of BALB/c mice to establish an orthotopic 4T1 tumor-bearing mouse model. When the tumor volume approached approximately 85 mm³, the mice were randomly divided into four groups (n = 5): Control, DOX·HCl, HFM, and HFMD. Following this, different treatments were administered through the tail vein based on the assigned groups. Throughout the experiment, we monitored both body weight and tumor volume. After the treatment period, 4T1 tumor-bearing mice were anesthetized with isoflurane to assess their cardiac function. Following this procedure, the mice were euthanized, allowing for the collection of tumors which were then stained with Tunel, Cleaved-caspase 3, and GPX4. Additionally, after the treatment, the main organs and blood samples from the mice were collected for pathological and biochemical analysis. DOX·HCl concentration of 5 mg/kg.

- **Evaluation of targeting ability of** **HFMD**

The HFM@DIR and DIR were administered to tumor-bearing mice via the tail vein. Subsequently, real-time imaging was performed using an *in vivo* imaging system. The mice were euthanized after 24 hours, and the tumor along with major organs (heart, lung, kidney, spleen, and liver) were collected, rinsed with cold saline, and subjected to fluorescent imaging to determine the biological distributions of HFM@DIR and DIR.

- **Measurement of DOX concentration in blood**

Prior to the experiment, SD rats were fasted for 12 hours without dehydration. The rats were divided into two groups (n=3): one receiving DOX·HCl and the other receiving HFMD, with a DOX·HCl concentration of 5 mg/kg. All samples were administered to the SD rats via the tail vein. Blood samples of 0.5 mL were collected from the orbital region at 10 minutes, 30 minutes, 1 hour, 1.5 hours, 2 hours, 4 hours, 8 hours, 18 hours, and 24 hours, and were placed in centrifuge tubes containing EDTA-2Na. The blood samples were centrifuged at 2500 rpm to separate the blood cells and obtain clear plasma. Subsequently, 200 μL of plasma was mixed with 1.8 mL of acidic methanol solution to precipitate insoluble substances. After centrifugation, the fluorescence intensity of the resulting clear solution was measured using a fluorescence spectrophotometer. By measuring the fluorescence intensity of the transparent solution obtained at various time points, the blood concentration-time curves for DOX·HCl and HFMD were established.

- **Biodistribution study**

The DOX·HCl and HFMD were administered intravenously to the tumor-bearing mice at a dosage of 5 mg/kg DOX·HCl (n=3). At 24 hours post-injection, the hearts, livers, spleens, lungs, kidneys, and tumors were harvested, then washed, weighed, and homogenized in saline using a tissue homogenizer. The resulting samples were combined with four volumes of acidic methanol and subsequently centrifuged at 12,000 rpm for a duration of 10 minutes. Fluorescence intensities of the supernatant were measured through fluorescence spectrophotometry.

- **Long-term toxicity test**

HFMD was administered to mice via tail vein injection at a dosage of 5 mg/kg, every two days for a total of five times (n=5). After 25 days, the mice were euthanized, and their organs were subjected to HE staining and biochemical analysis tests.


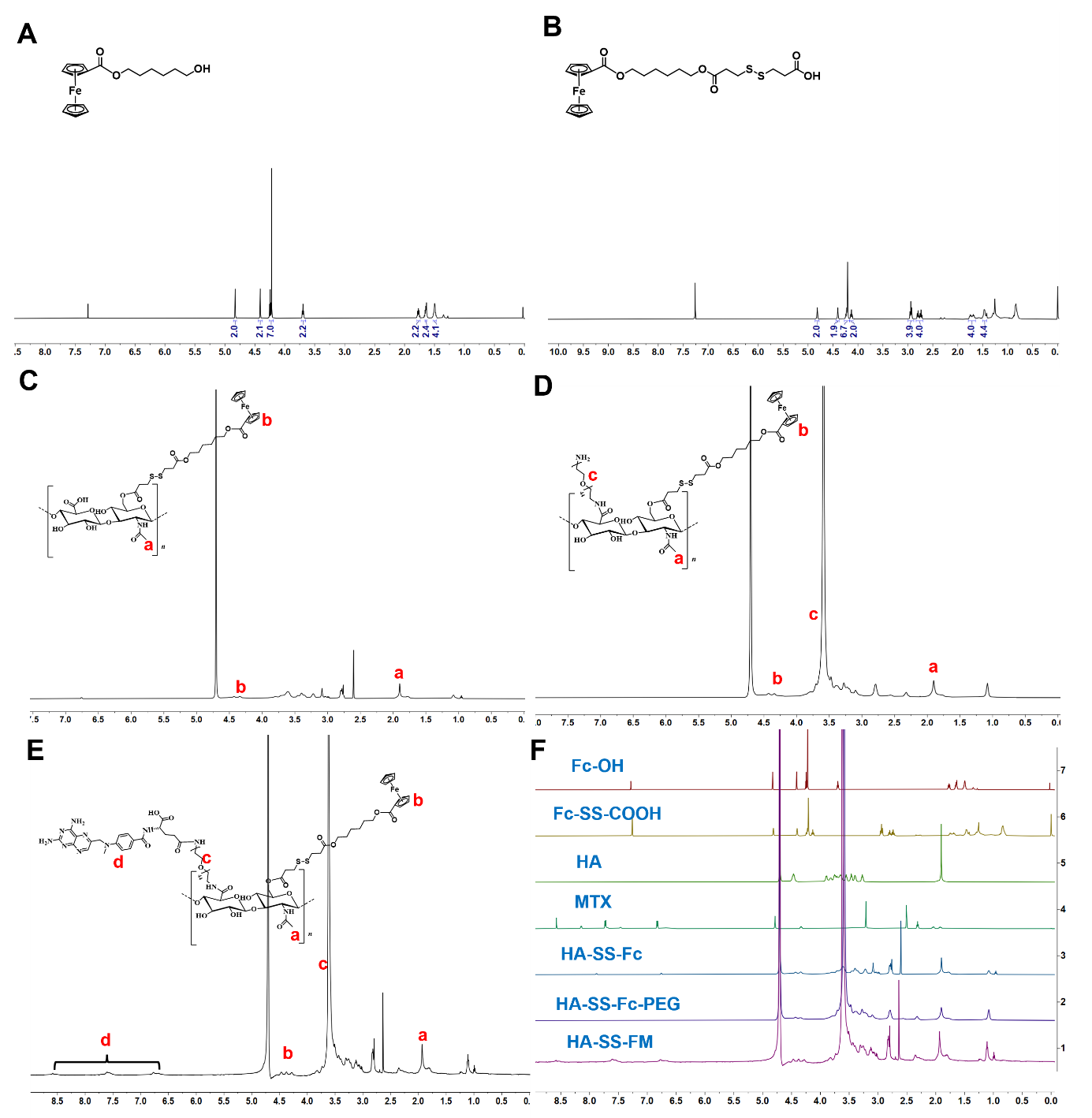


**Fig. S1** ^1^H-NMR spectra of (A) Fc-OH, (B) Fc-SS-COOH, (C) HA-SS-Fc, (D) HA-SS-Fc-PEG, (E) HA-SS-FM. Overlay of Fc-OH, Fc-SS-COOH, HA, MTX, HA-SS-Fc, (D) HA-SS-Fc-PEG and HA-SS-FM ^1^H NMR spectrum.


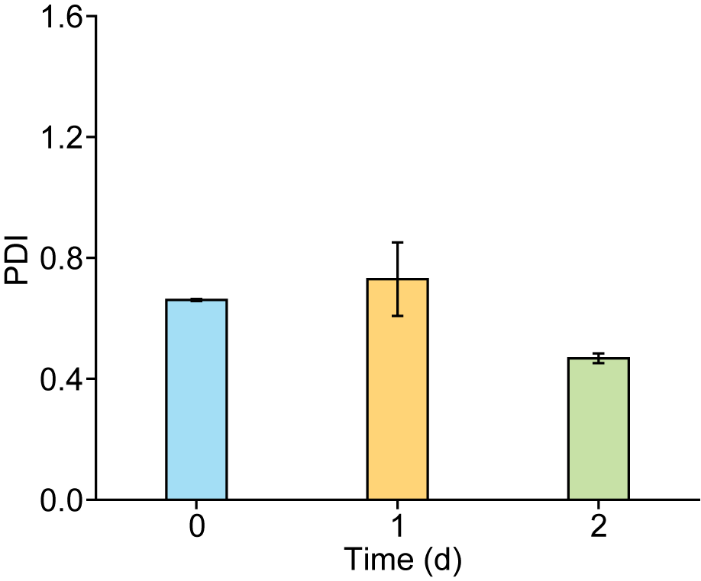


**Fig. S2** PDI of HFMD in 10% FBS environment. **
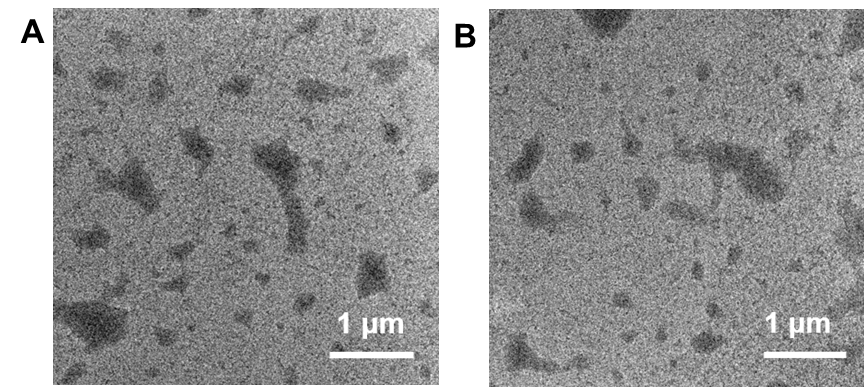
**

**Fig. S3** TEM image of HFMD under high GSH (A) and ROS (B) conditions.


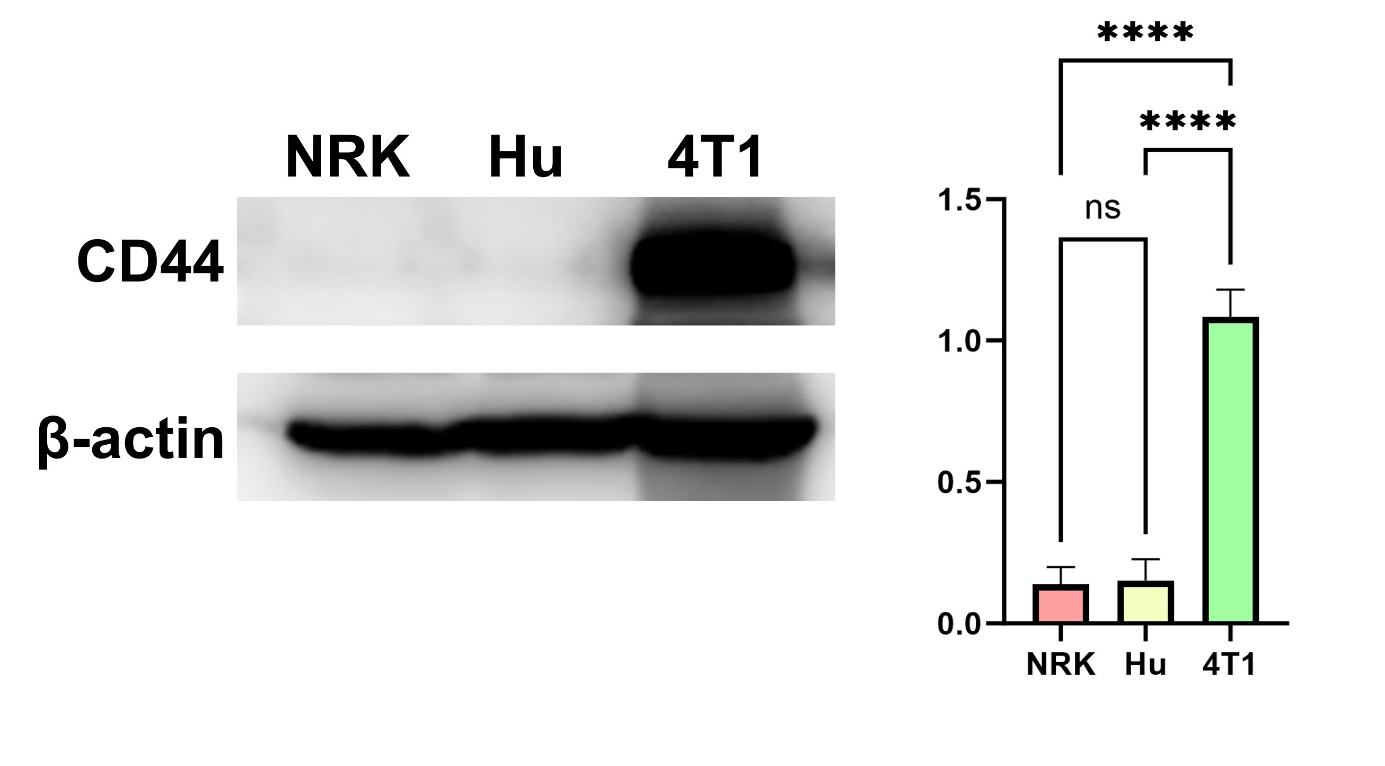


**Fig. S4** CD44 protein expression levels in NRK-49F, HUVECS, and 4T1 cells. *****p*< 0.0001.


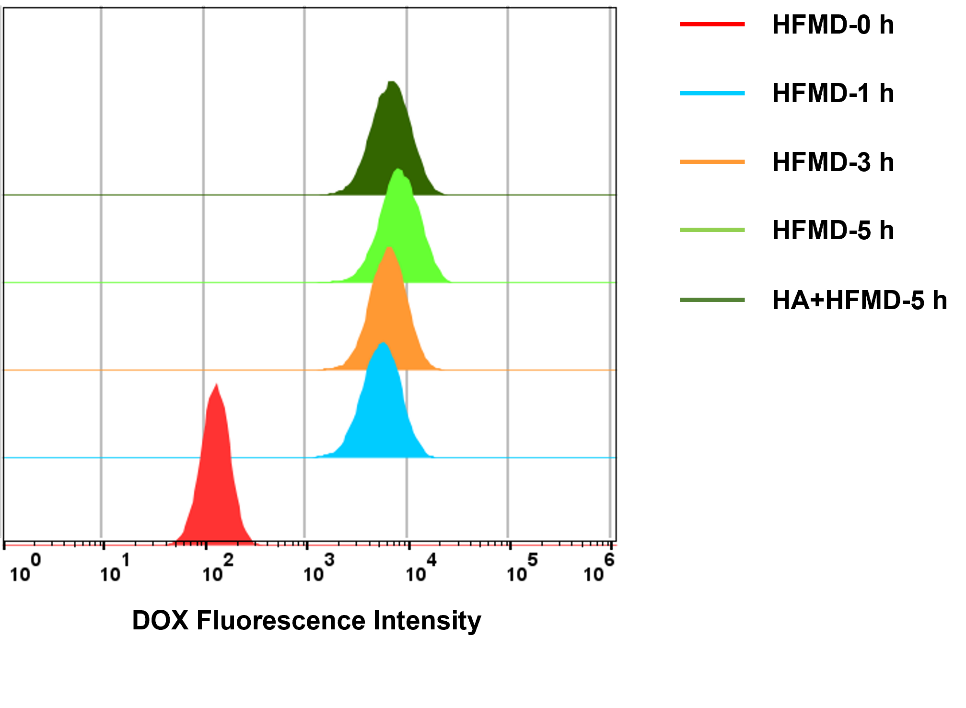


**Fig. S5** Flow cytometry images of HFMD and HA+HFMD uptake by 4T1 cells at different time periods.


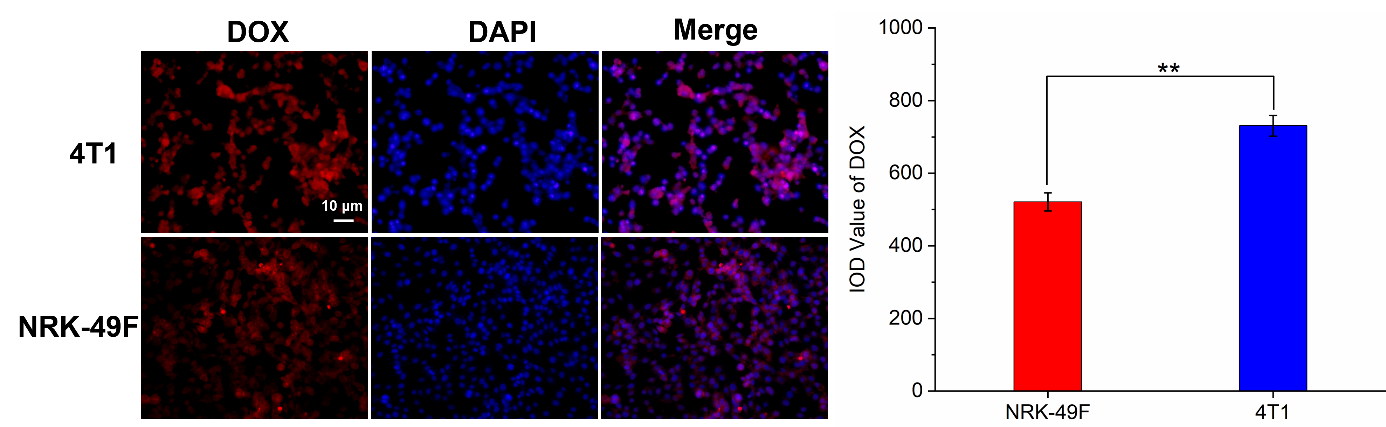


**Fig. S6.** The inverted fluorescence microscopy images of NRK-49F cells and 4T1 cells after 5 hours of HFMD uptake. ***p*< 0.001.


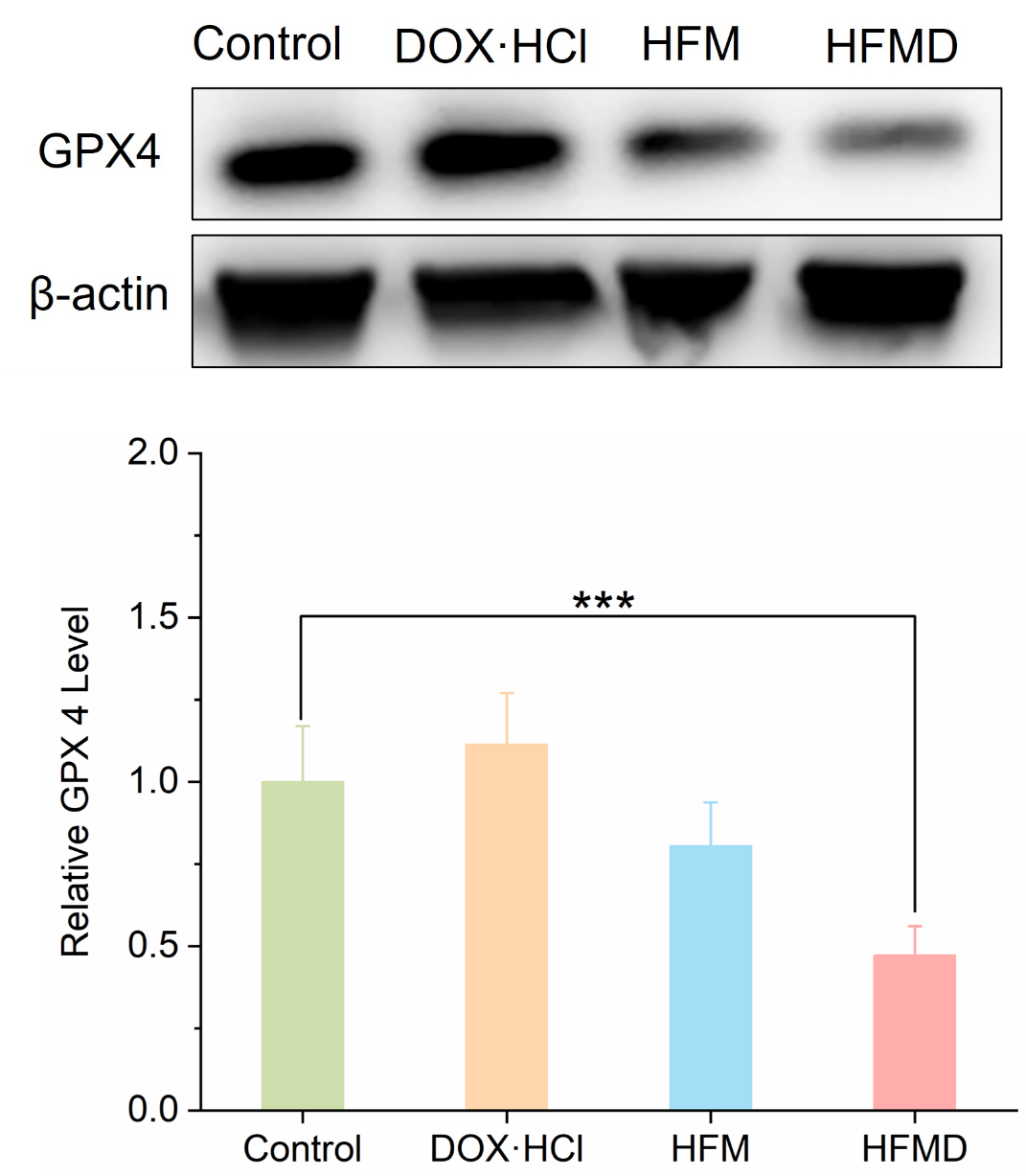


**Fig. S7** After incubating with various preparations for 24 h, the protein levels of GPX4**.** ****p*< 0.001.


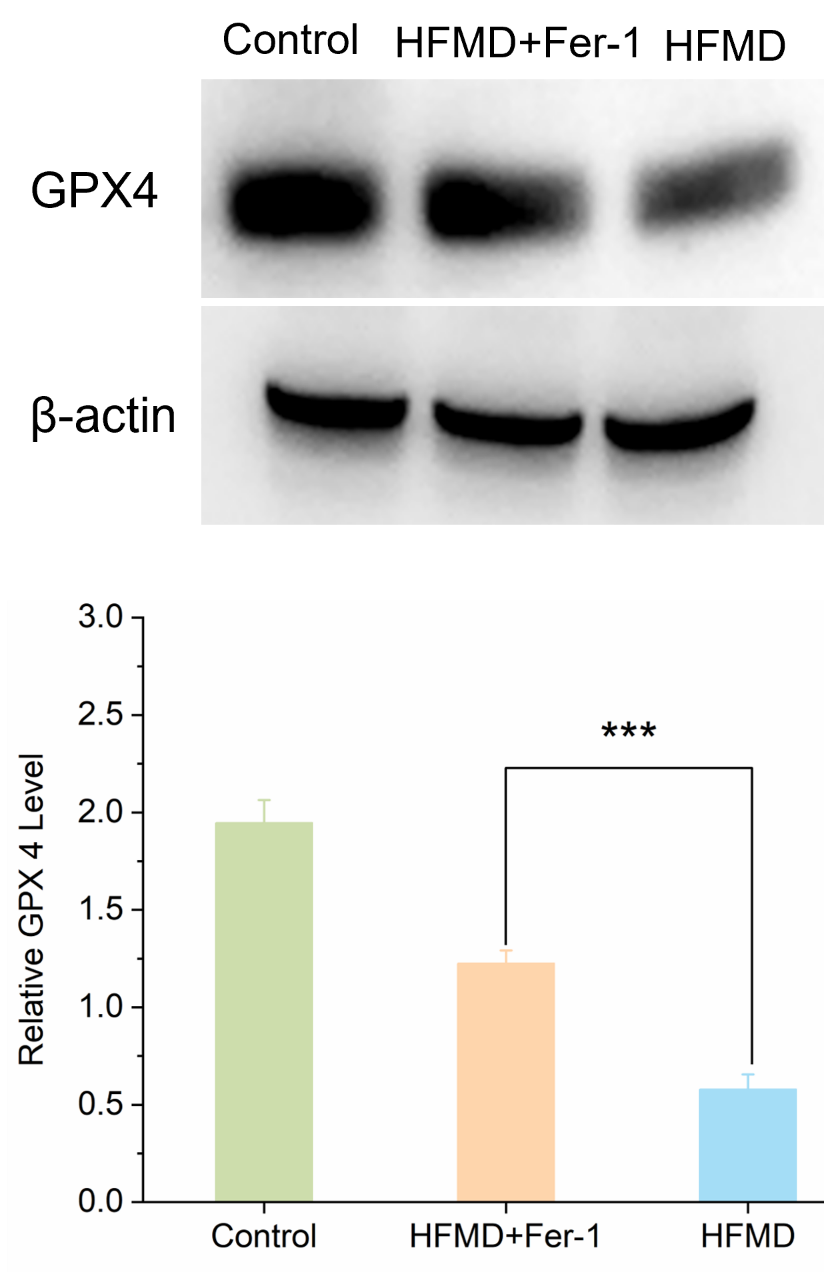


**Fig. S8** After incubating with HFMD+Fer-1 and HFMD for 24 h, the protein expression level of GPX4. ****p*< 0.001.


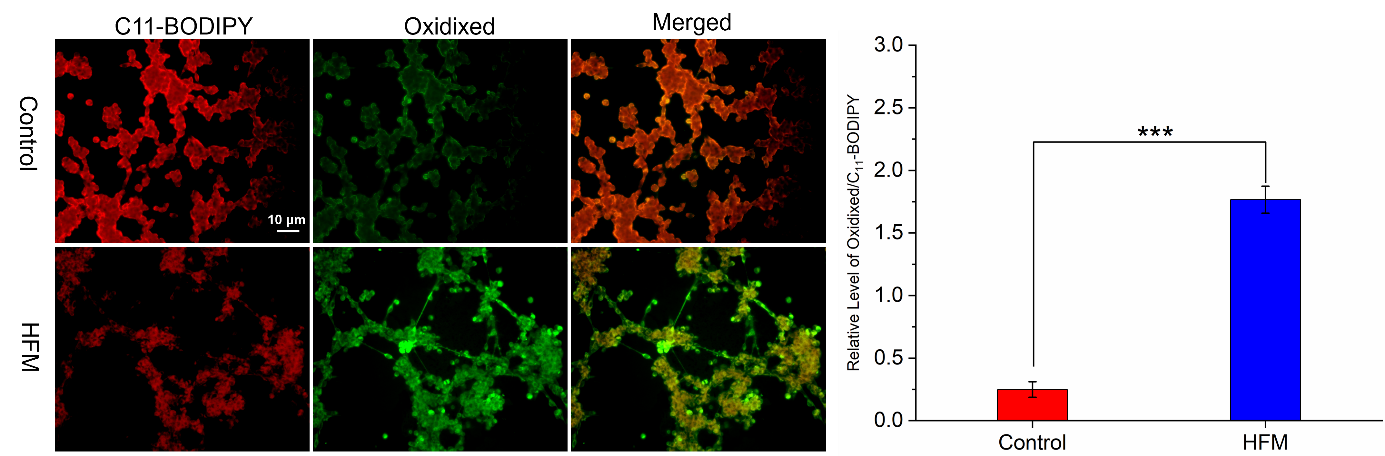


**Fig. S9** The fluorescent inverted microscope images of lipid peroxidation generation in 4T1 cells treated with HFM. ****p*< 0.001.


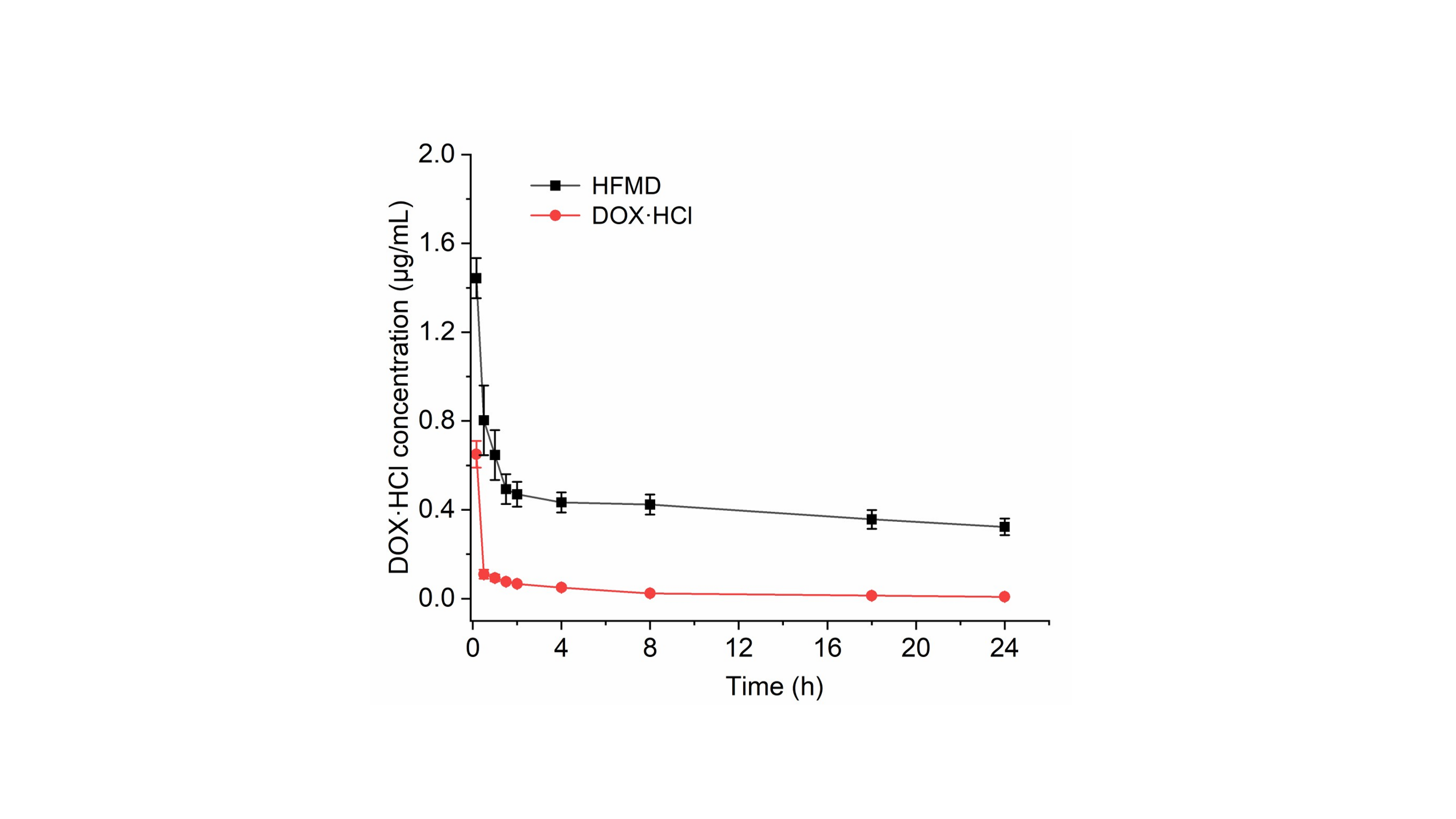


**Fig. S10** Drug concentration-time curves of DOX·HCl and HFMD.


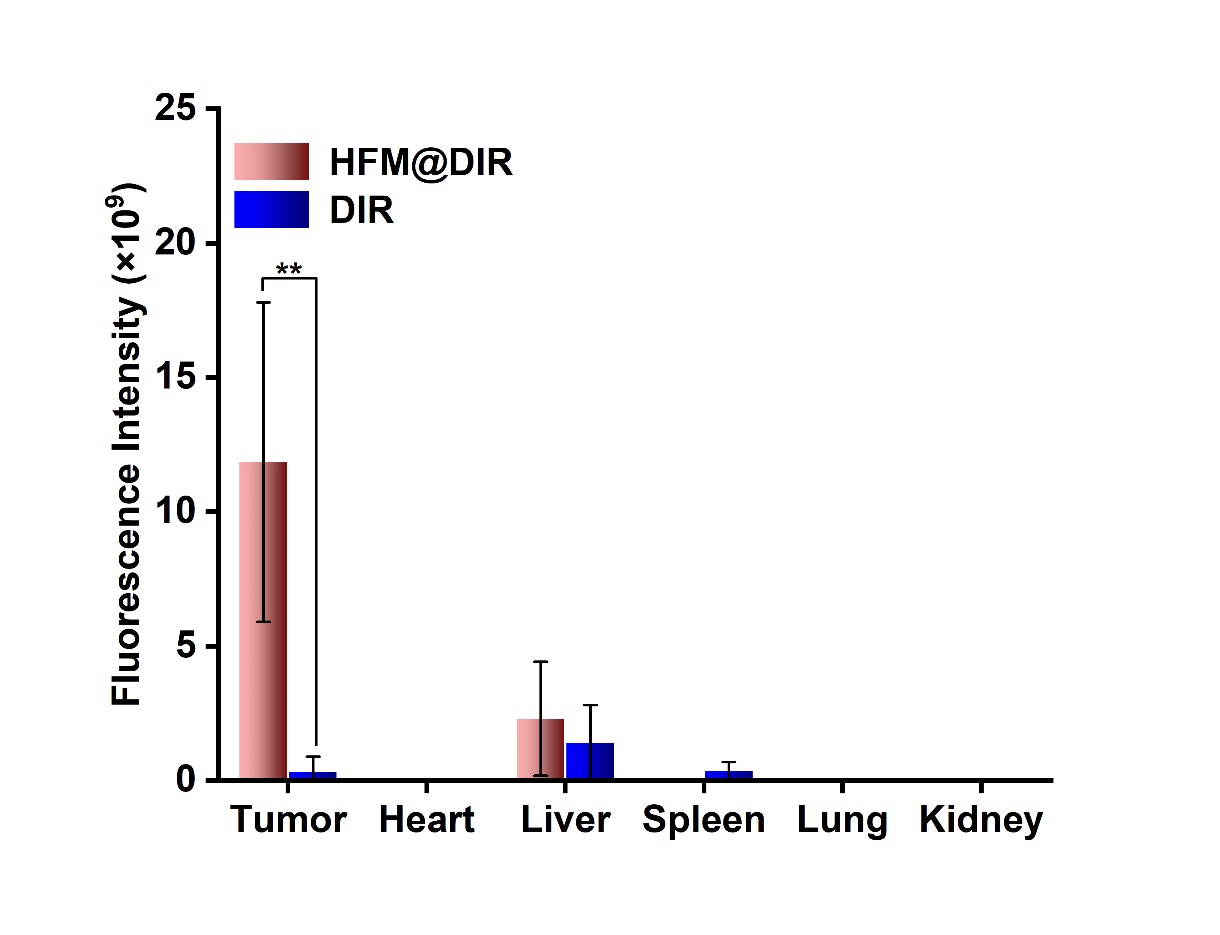


**Fig. S11** Fluorescence imaging of major organs and tumors of mice in HFM@DIR and DIR groups after 24 h. ***p*< 0.01.


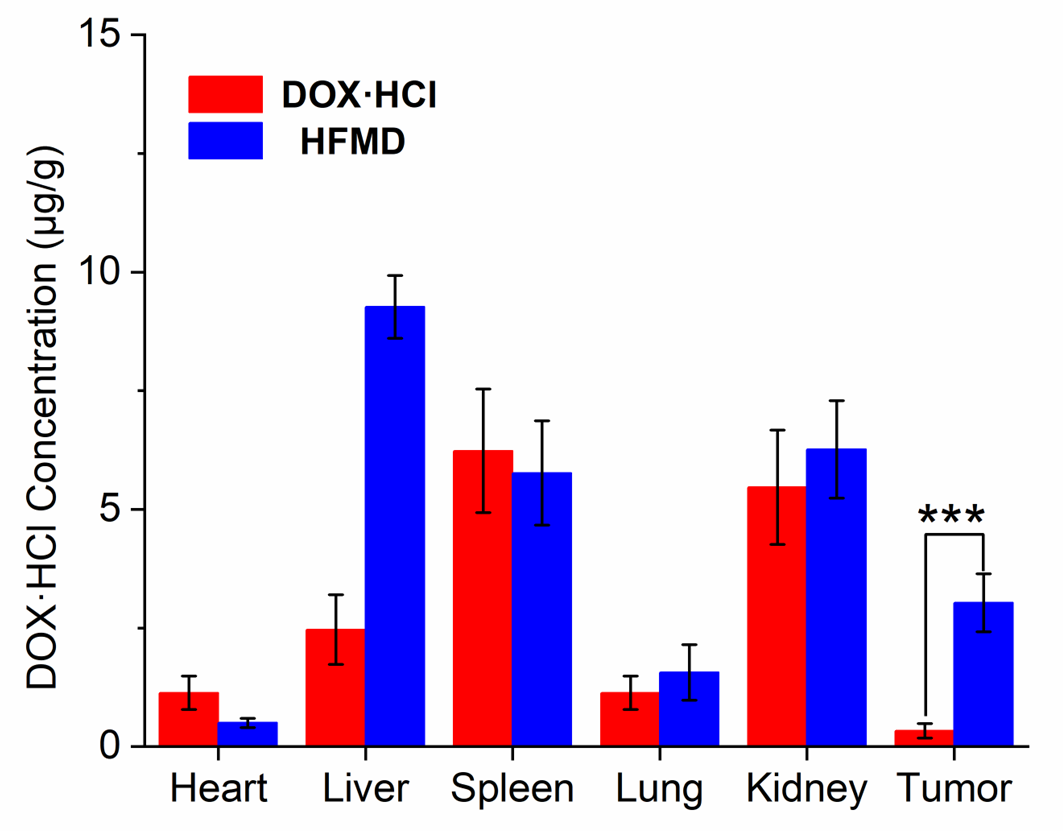


**Fig. S12** Twenty-four hours after the injection of DOX·HCl and HFMD, the *in vivo* distribution of DOX in 4T1 tumor-bearing mice. ****p*< 0.001.


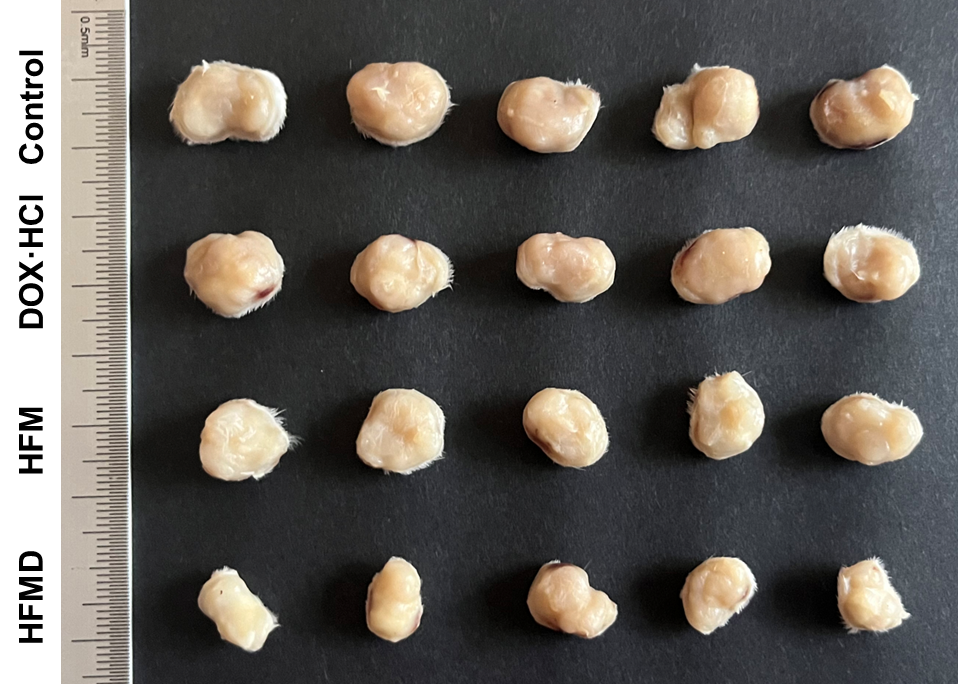


**Fig. S13** The tumor comparison after 12 days of treatment.





**Fig. S14** Changes in body weight of tumor-bearing mice in various treatment groups. ****p*< 0.001.


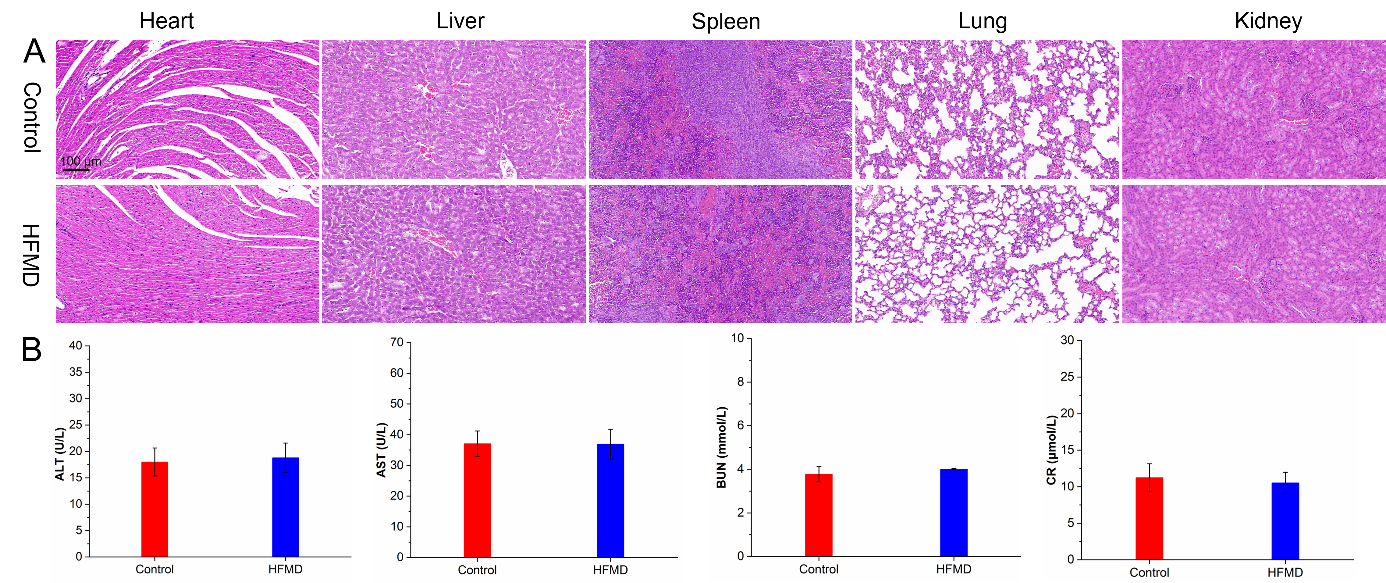


**Fig. S15** The tail vein injection of HFMD was administered five times (once every two days), and after 25 days, HE staining of various organs (A) and biochemical values (B) in each group of mice.
